# The APSES transcription factor Swi6B upregulates *CATALASE 1* transcription to enhance oxidative stress tolerance of *Ganoderma lucidum*

**DOI:** 10.1128/aem.00679-25

**Published:** 2025-06-18

**Authors:** Lingshuai Wang, Lingyan Shi, Shuhan Zhang, Jiping Ma, Cheng Zhang, Huhui Chen, Mingwen Zhao

**Affiliations:** 1 Key Laboratory of Agricultural and Environmental Microbiology, Ministry of Agriculture and Rural Affairs, College of Life Sciences, Nanjing Agricultural University 70578, Nanjing, China; 2 Institute of Agricultural Applied Microbiology, Jiangxi Academy of Agricultural Sciences 205386, Nanchang, China; Chalmers tekniska hogskola AB, Gothenburg, Sweden

**Keywords:** *Ganoderma lucidum*, oxidative stress, Swi6B, *CAT1*

## Abstract

As sessile organisms, fungi often encounter various stresses throughout their lifespan, resulting in the overproduction of reactive oxygen species, which impedes the normal growth of fungi. Previously, we revealed that the Swi6B transcription factor is involved in the stress tolerance of *Ganoderma lucidum*. However, the underlying molecular mechanism is unclear. The present study demonstrated that oxidative stress increased the levels of Swi6B. Direct binding of Swi6B to the promoter region of *catalase 1* (*CAT1*) resulted in increased transcription of *CAT1* and reduced H_2_O_2_ levels in *SWI6B* overexpressing strains. In addition to increased Swi6B protein, the phosphorylation level of Swi6B was increased by H_2_O_2_ treatment. The overexpression of *SLT2*, a mitogen-activated protein kinase that interacts with Swi6B, increased the phosphorylation level of Swi6B, and treatment with H_2_O_2_ further enhanced this increase. As a result, both the binding of Swi6B to the *CAT1* gene and tolerance to H_2_O_2_ were increased in *SLT2*-overexpressing strains. The present findings revealed that the Slt2-Swi6B-CAT1 pathway responds to oxidative stress and contributes to improving the survival of *G. lucidum* in adverse environments.

**IMPORTANCE:**

In fungi, environmental stress leads to the accumulation of intracellular reactive oxygen species and leads to oxidative stress. Here, we found that the overexpression of the APSES transcription factor *Swi6B* enhances tolerance to oxidative stress in *Ganoderma lucidum*. Swi6B binds to the promoter region of *CAT1*, which increases *CAT1* transcription and reduces the H_2_O_2_ levels. In addition, the phosphorylation of Swi6B by Slt2 promotes the regulation of *CAT1* by Swi6B. The Slt2-Swi6B-CAT1 pathway is important for the response of *G. lucidum* to oxidative stress.

## INTRODUCTION

Fungi can perceive and adapt to various external stress conditions (such as heat, drought, and heavy metals) encountered in a dynamically changing growth environment (1, 2). With continuous exposure to these stressors, the levels of harmful chemicals, including reactive oxygen species (ROS), within the cytoplasm of fungi significantly increase, which results in irreversible damage to DNA, proteins, lipids, and other cellular components, ultimately leading to cell death (3, 4). Therefore, managing and eliminating high levels of ROS induced by external stress is important for the survival of fungi (5).

One fundamental feature of normal physiological metabolic activity in living organisms is the generation of ROS, which consist of molecular oxygen (6). These metabolic activities occur mainly in mitochondria and peroxisomes. Superoxide (O_2_·^−^), hydroxyl radical (•OH), and hydrogen peroxide (H_2_O_2_) are the three main types of ROS, and they have important physiological functions (7). Low levels of ROS are essential for cellular activities, whereas high levels of ROS lead to irreversible damage to cellular components (8–10). Elevated levels of ROS have the potential to induce DNA breaks and base mutations, as well as regulate the interaction between proteins and DNA (11). Increased ROS levels can also disrupt the proper arrangement of the phospholipid bilayer by promoting lipid oxidation in the cytoplasm, resulting in denaturation and inactivation of certain receptors and enzymes bound to the cell membrane, as well as an increase in tissue permeability (12). Moreover, elevated levels of ROS can result in the fragmentation of peptide chains, alterations in the charge properties of proteins, and a reduction in the activity or degradation of specific proteolytic enzymes (5).

Naturally, organisms have developed specific mechanisms to counteract ROS bursts caused by environmental stress (13–15). Mitogen-activated protein kinase (MAPK) pathways are evolutionarily conserved regulators of the fungal oxidative stress response. In model fungi, distinct MAPK modules orchestrate redox homeostasis through specialized roles. In fission yeast, Sty1p (MAPK) phosphorylation increases with increasing H_2_O_2_ levels, and its deletion severely impairs oxidant resistance and survival under moderate oxidative stress (16). Similarly, *Ganoderma lucidum Slt2* (MAPK)-knockdown strains exhibit hypersensitivity to oxidation and growth inhibition (17). In *Phytophthora sojae*, H_2_O_2_ induces the expression of the *PsMPK7* MAPK homolog, which is essential for scavenging host-derived ROS and maintaining pathogenicity (18). The *Bipolaris oryzae SRM1* (MAPK) mutant (Δ*srm1*) displays H_2_O_2_-sensitive growth, which is linked to its role in activating *catalase 2* expression to increase oxidative stress tolerance (19). While these studies underscore MAPK-mediated ROS defense, mechanistic insights into how MAPK cascades coordinate with downstream antioxidant systems (e.g., catalases) remain unclear.

In cells, catalase (CAT) directly reduces H_2_O_2_ to H_2_O and O_2_ within the peroxisome and thus plays a pivotal role in response to oxidative stress by maintaining intracellular ROS homeostasis (20). Heat stress is one of the key factors that affect the growth and development of fungi (21), and heat stress induces an increase in ROS in fungi (22). *CAT* overexpression enhances strain growth at high temperatures, whereas *CAT* deletion dramatically inhibits strain growth at high temperatures (23). Deletion of *CAT1* in *Aspergillus flavus* results in a significant increase in intracellular ROS levels, and the *Δcat1* mutant exhibits markedly increased sensitivity to H_2_O_2_-induced oxidative stress (20). When H_2_O_2_ is added exogenously to the culture medium of *Aspergillus oryzae*, the expression of *CATB,* which encodes catalase, is significantly induced (24). Similarly, the survival rate of *Aspergillus nidulans CATB* mutant spores is significantly reduced after treatment with different concentrations of H_2_O_2_, and all *ctaB* mutant spores are killed when the H_2_O_2_ concentration reaches 20 mM (25). These findings suggest that *CAT* plays a crucial role in ROS scavenging and serves as a central player in the defense against oxidative stress.

Previously, we reported that reducing the expression of *SWI6B*, a transcription factor downstream of the MAPK signaling pathway in *G. lucidum*, significantly decreases the tolerance of knockdown strains to exogenous H_2_O_2_ (26). Further studies have revealed that the expression level of *SWI6B* increases after oxidative stress treatment. To study the functional mechanism underlying the enhanced resistance of Swi6B to oxidative stress, we analyzed the gene expression and activity of the *G. lucidum* ROS scavenger. The present results demonstrated that Swi6B directly binds to the promoter of *CAT1* to upregulate the transcription of *CAT1* and thus increase the ability of *G. lucidum* to scavenge ROS, improving tolerance to oxidative stress.

## RESULTS

### Swi6B responds to and enhances the tolerance of *G. lucidum* to oxidative stress

**Fig 1:**
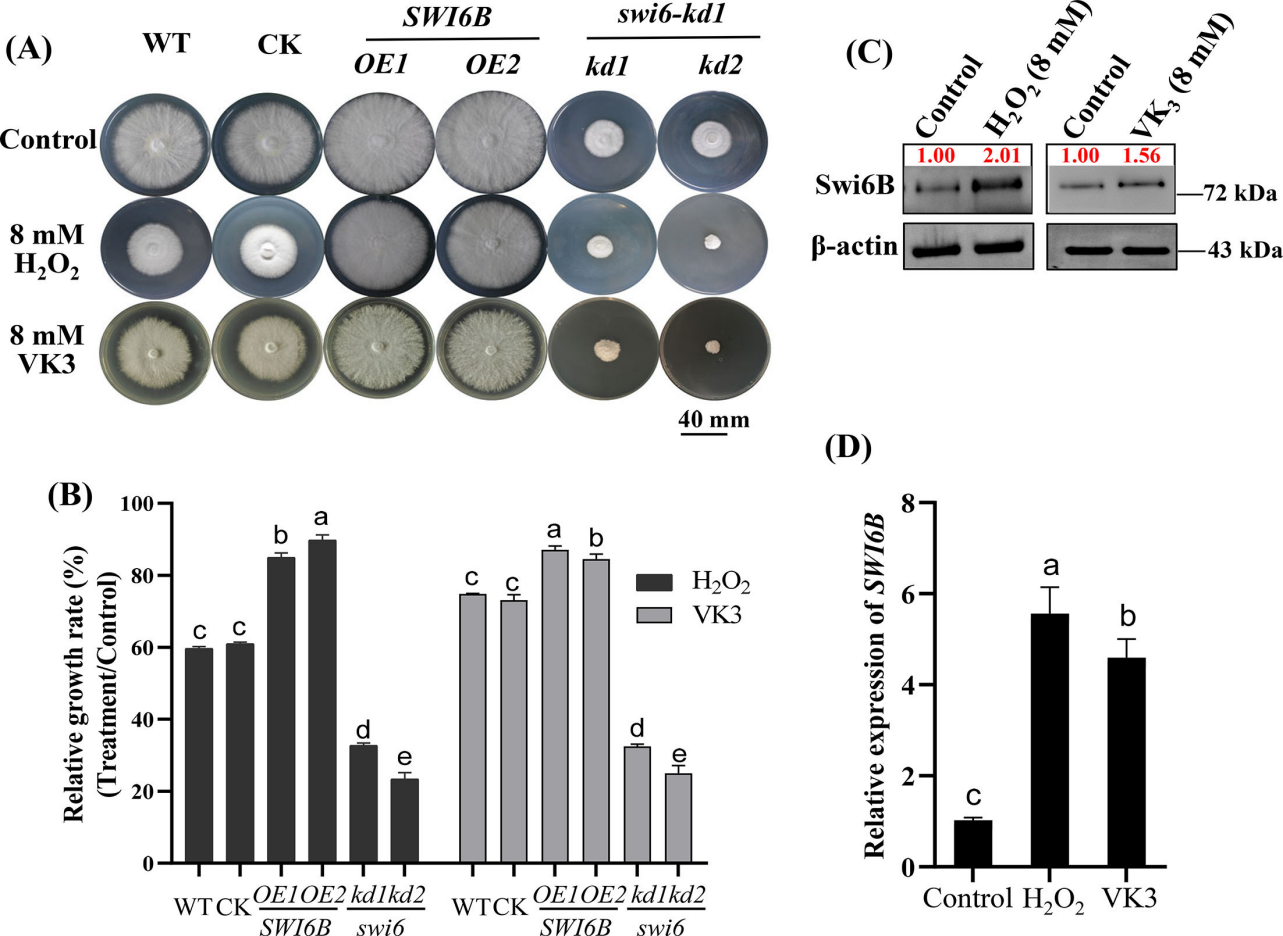
Swi6B responses and enhances the tolerance of *G. lucidum* to oxidative stress. (**A and B**) The representative pictures (**A**) and relative growth rates (**B**) of different genotype strains under oxidative stressor treatment. All strains were cultured on CYM solid medium supplemented with 8  mM H_2_O_2_ or 8  mM VK_3_. The relative growth rate of each strain was calculated as the diameter of hyphae growth under H_2_O_2_ or VK_3_ treatment divided by that under control condition. The data were presented as a percentage. *SWI6B-OE*, *SWI6B* overexpression strains; *swi6-kd*, *SWI6* knockdown strains; CK, empty vector strains. (**C**) Western blotting analysis of the Swi6B protein in the WT strain grown on CYM solid medium supplemented with or without oxidant. The intensity of bands was analyzed with Image J software (v1.8.0). (**D**) Quantitative real-time PCR analysis of the expression levels of *SWI6B* in the WT strain cultured with or without oxidant treatment. For panels **B** and **D**, the different letters indicate significant differences according to Duncan’s multiple range test (*P*  <  0.05). For panels **C** and **D**, the values of control were set to 1.00 to normalize the values under H_2_O_2_ (8  mM) or VK_3_ (8  mM) treatment.

Previous studies have shown that the *SWI6* gene undergoes alternative splicing and produces two isoforms, namely, *SWI6A* and *SWI6B* (26, 27). Here, the growth of *SWI6* knockdown strains (*swi6-kd*) was repressed, and these strains were hypersensitive to H_2_O_2_ and menadione (VK_3_) treatment (Fig. 1A and B). To further study whether *SWI6* is involved in response to oxidative stress, both the *SWI6A-* and *SWI6B*-overexpressing strains (*SWI6A-OE* and *SWI6B-OE*) (27) were treated with H_2_O_2_ and VK_3_. Compared with that of the wild-type (WT) strain, the growth of *SWI6B*-overexpression strains was significantly greater under H_2_O_2_ and VK_3_ treatment conditions (Fig. 1A and B), whereas the growth of *SWI6A*-overexpression strains was similar to that of the WT strain (Fig. S1A and B). Furthermore, both H_2_O_2_ and VK_3_ treatment induced dramatically increased *SWI6B* transcription and protein levels (Fig. 1C and D) but did not significantly affect the transcription and protein levels of *SWI6A* (Fig. S1C and D).

### Swi6B increases *CAT1* expression to reduce H_2_O_2_ content

**Fig 2:**
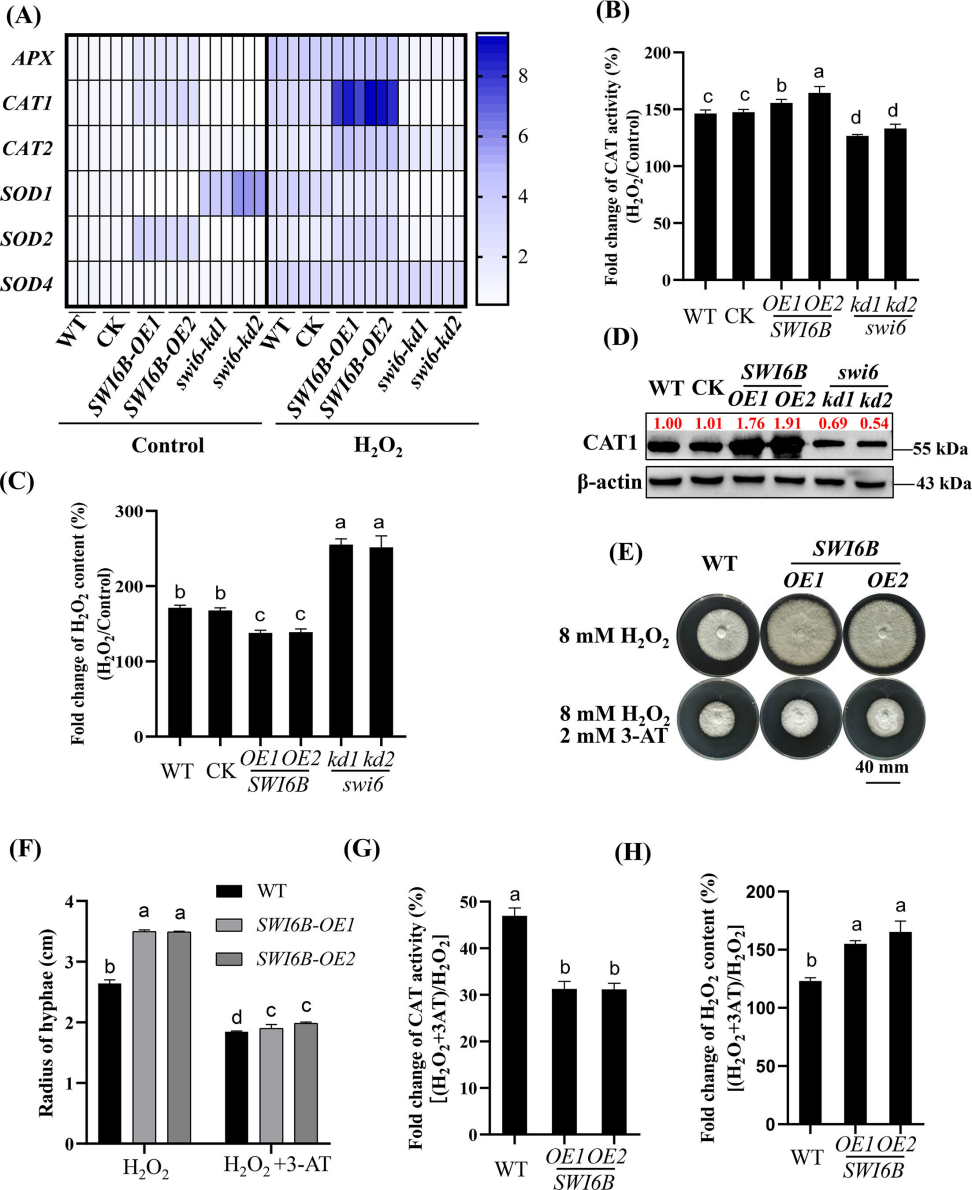
Overexpression of *SWI6B* promotes the expression and enzyme activity of CAT1 to reduce H_2_O_2_ content. (**A**) Heat map of the expression levels of genes encoding classical antioxidant enzymes with or without H_2_O_2_ treatment. The expression levels of genes in all the genotypes were analyzed by RT-qPCR. (**B**) Western blotting analysis of the CAT1 protein in the *SWI6B-OE*, *swi6-kd*, WT, and CK strains. All strains grown on CYM solid medium were added with or without H_2_O_2_. The intensity of bands was analyzed with Image J software (v1.8.0), and the values of WT treated without H_2_O_2_ were set to 1.00. (**C and D**) Fold change of relative CAT activity (**C**) or the H_2_O_2_ content (**D**) in different genotype strains cultured under H_2_O_2_ treatment. The relative enzyme activity or H_2_O_2_ content of each strain was calculated as the CAT activity or H_2_O_2_ content in the presence of H_2_O_2_ divided by the control, respectively. (**E and F**) The representative pictures (**E**) and hyphae radius (**F**) of *SWI6B-OE* and WT strains cultured on H_2_O_2_ (8 mM) containing CYM solid medium supplemented with or without 3-amino-1,2,4-triazole (3-AT; 2 mM). (**G and H**) Fold change of relative CAT activity (**G**) or the H_2_O_2_ content (**H**) in *SWI6B-OE* and WT strains. All strains were cultured as described (**E**). The relative enzyme activity or H_2_O_2_ content of each strain was calculated as the CAT activity or H_2_O_2_ content in the presence of H_2_O_2_ + 3-AT divided by that under *SWI6B-OE* and WT, respectively. For panels **C, D**,** F, and H**, the different letters indicate significant differences according to Duncan’s multiple range test (*P* < 0.05).

To understand the mechanism underlying the enhanced tolerance of *SWI6B* to oxidants, the expression levels of several antioxidant enzyme-encoding genes (*APX*, *CAT1*, *SOD1*, and *SOD2*) in different strains (WT, *SWI6B-OE*, and *swi6-kd*) were analyzed via quantitative real-time PCR (qRT-PCR). Under control conditions, the expression levels of *APX*, *CAT1*, and *SOD2* were greater in the *SWI6B-OE* strains than in the WT strain (Fig. 2A). Compared with the control conditions, H_2_O_2_ treatment significantly increased the transcription of *APX* and *CAT1* (Fig. 2A). Notably, after H_2_O_2_ treatment, the expression level of *CAT1* was the highest in the *SWI6B-OE* strains, while there was no significant difference in the expression level of *CAT1* in the *swi6-kd* strains (Fig. 2A). In addition, the protein levels of CAT1 in different strains were detected by western blot analysis with a specific CAT1 antibody. As shown in Fig. 2D, the protein levels of CAT1 were significantly increased in the *SWI6B-OE* strains (1.76–1.91) but significantly reduced in the *swi6-kd* strains (0.54–0.69). These findings suggested that Swi6B plays an important role in mediating H_2_O_2_-induced *CAT1* expression.

Subsequently, we measured the H_2_O_2_ content and antioxidant enzyme activity in strains with or without H_2_O_2_ treatment. The results revealed that the increase in the endogenous H_2_O_2_ content induced by H_2_O_2_ treatment was minimal in the *SWI6B-OE* strains but maximal in the *swi6-kd* strains (Fig. 2B), suggesting a significantly greater ability to eliminate H_2_O_2_ in the *SWI6B-OE* strains than in the WT strains. Consistent with this finding, after H_2_O_2_ treatment, the CAT activity in the *SWI6B-OE* strains significantly increased but obviously decreased in the *swi6-kd* strains (Fig. 2C). Although other genes were differentially regulated in the engineered strains (Fig. 2A), no significant change was detected in the activities of the superoxide dismutase (SOD) and ascorbate peroxidase (APX) enzymes in any of the genotype strains (Fig. S2C and D). To further verify the CAT1-dependent H_2_O_2_ tolerance of the *SWI6B-OE* strains, the 3-amino-1,2,4-triazole (3-AT) catalase inhibitor was used in the treatment experiments. With H_2_O_2_ treatment alone, the growth of the *SWI6B-OE* strains was obviously better than the WT strain (Fig. 2E and F). However, when the strains were grown on the media supplemented with both H_2_O_2_ and 3-AT, the growth rates of the WT and SWI6B-OE1/2 strains were similar (Fig. 2E and F), indicating that 3-AT had a more pronounced inhibitory effect on the growth of the *SWI6B-OE* strains (46% and 44%) compared to the WT strain (31%). Moreover, the CAT activity and H_2_O_2_ content of the strains treated with or without 3-AT were evaluated. Following the exogenous H_2_O_2_ and 3-AT treatments, the CAT activity in all the strains was lower than that in the H_2_O_2_-only treatment. However, after the addition of 3-AT, the fold change in CAT activity in the *SWI6B-OE* strains was obviously lower than that in the WT strain (Fig. 2G). Consistent with these findings, compared with the WT, H_2_O_2_ content in the *SWI6B-OE* strains was significantly greater (Fig. 2H). These results suggested that Swi6B enhances H_2_O_2_ tolerance by increasing *CAT1* transcription and protein accumulation. In addition, although the overall expression level of *CAT2* is lower than that of *CAT1*, the response of *CAT2* to H_2_O_2_ is similar to that of *CAT1*. Thus, we hypothesize that Swi6B may indirectly regulate *CAT2* transcription.

### Swi6B directly binds to the promoter of *CAT1* to upregulate its transcription

**Fig 3:**
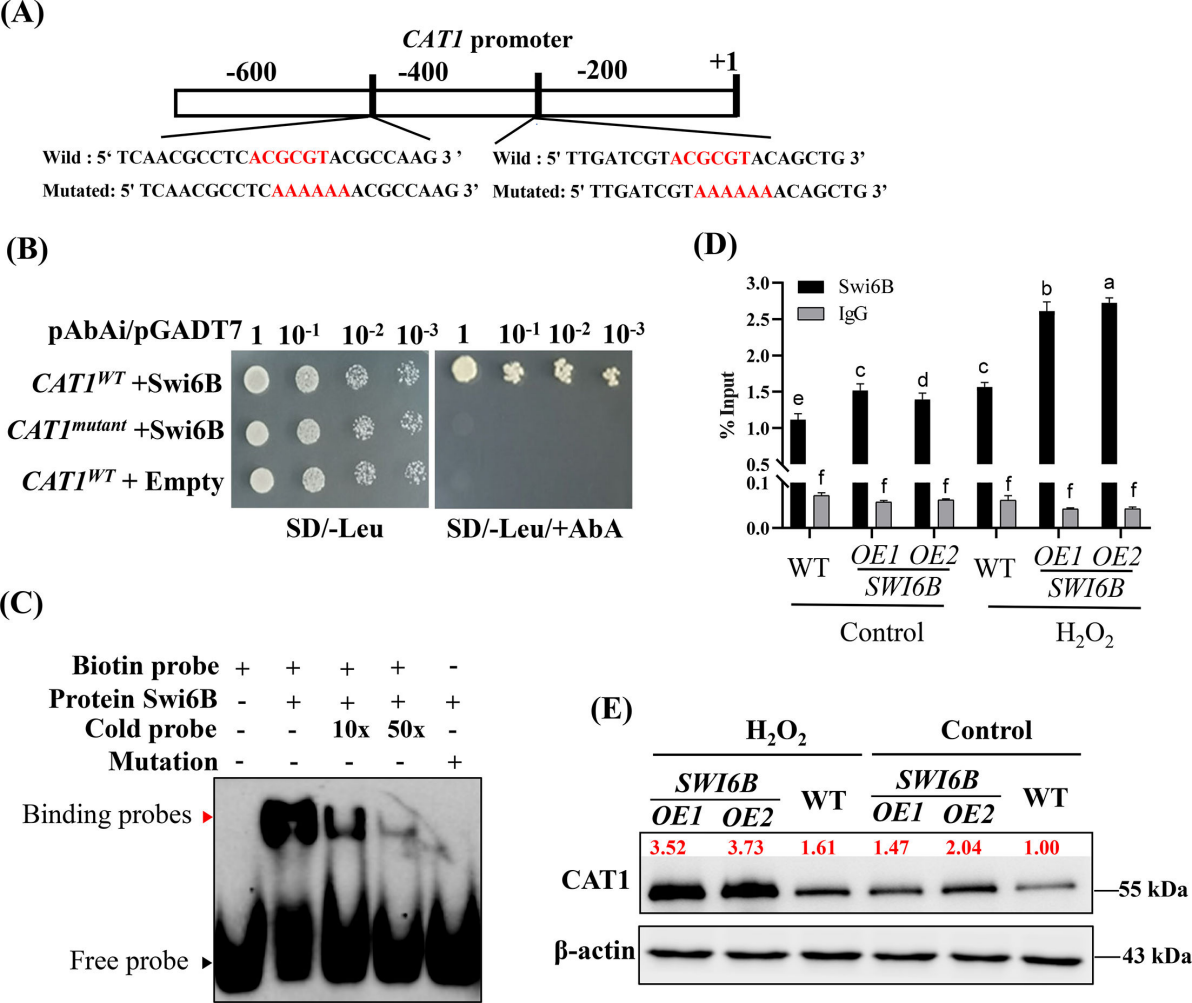
H_2_O_2_ treatment improves the binding of Swi6B with the promoter of *CAT1* to promote CAT1 accumulation. (**A**) Schematic diagram of the Swi6B binding elements (*CAT1^WT^*) and mutation elements (*CAT1^mutant^*) present in the *CAT1* promoter region. (**B and C**) Y1H (**B**) and EMSA assays exhibited the interaction between Swi6B and the *CAT1 ^WT^* but not the *CAT1^mutant^* fragments. (**D**) ChIP-qPCR analysis showed the binding of Swi6B to the promoter of *CAT1* in the *SWI6B-OE* and WT strains treated with or without H_2_O_2_. The different letters indicate significant differences according to Duncan’s multiple range test (*P*  <  0.05). (**E**) Western blotting analysis of the H_2_O_2_-induced CAT1 protein in the *SWI6B-OE* and WT strains. All strains grown on CYM solid medium supplemented with or without H_2_O_2_. The intensity of bands was analyzed with Image J software (v1.8.0), and the values of WT under control condition were set to 1.00.

As an APSES transcription factor, Swi6B performs physiological functions by activating gene expression (27). Owing to the obvious change in the transcription of antioxidant genes, we investigated the cis-regulatory elements bound by Swi6B in these genes. Analysis of the promoter sequences (~1,000 bp) of genes encoding SOD, CAT, and APX via the JASPAR database (https://jaspar.elixir.no/). The results exhibited that the promoter of *CAT1* contains two conserved MluI cell cycle box (MCB)-binding elements (ACGCGT), which are binding sites specific for the APSES transcription factor family, at 364-370 bp and 445-451 bp upstream of the start codon (Fig. 3A). Although H_2_O_2_ treatment increased the expression levels of both *CAT1* and *CAT2* in the WT and *SWI6B-OE* strains, only the promoter of *CAT1* contained the MCB-binding element of Swi6B. Thus, we focused on the study of *CAT1*. Moreover, this specific element was not found in the upstream promoter motifs of the *APX*- and *SOD*-encoding genes. Different experiments were subsequently conducted to further verify the interaction between Swi6B and the MCB-binding element. In the yeast one-hybrid (Y1H) assay, Swi6B interacts with the fragment containing two natural MCB-binding elements but did not bind to the mutated MCB-binding element (Fig. 3B). Swi6B and biotin-labeled probes from the *CAT1* promoter region were used in the electrophoretic mobility shift assay (EMSA) to further investigate the specific interactions between Swi6B and the MCB-binding element. As shown in Fig. 3C, the addition of Swi6B resulted in a clearly shifted band of the labeled probe, whereas no shifted band was observed when mutated probes were added. Additionally, as the concentration of the unlabeled probes increased, the intensity of the shifted band significantly decreased. These results suggest that Swi6B specifically binds to the MCB-binding element within the promoter region of *CAT1*. To further validate these findings, chromatin immunoprecipitation (ChIP)-qPCR was performed to confirm the interaction between Swi6B and the MCB-binding element. Under normal conditions, the enrichment of *CAT1* in the *SWI6B-OE* strains was approximately 1.50-fold greater than that in the WT strain (Fig. 3D). Although the enrichment of *CAT1* was increased after H_2_O_2_ treatment, the degree of induction in the *SWI6B-OE* strains was obviously greater than that in the WT strain (Fig. 3D). Together, these results suggested that Swi6B directly binds to the promoter of *CAT1* to activate the transcription of *CAT1*, and H_2_O_2_ treatment enhances this binding. To further confirm these results, the protein levels of CAT1 in the WT and *SWI6B-OE* strains were quantified with a CAT1 antibody. Consistent with the increase in transcription, H_2_O_2_ treatment also increased the level of the CAT1 protein in the WT and *SWI6B-OE* strains (Fig. 3E). After H_2_O_2_ treatment, the CAT1 protein levels in the *SWI6B-OE* strains increased by 2.32-fold, compared with that in the H_2_O_2_-treated WT strain. Together, these data demonstrated that Swi6B can bind to the *CAT1* promoter to promote the expression levels of *CAT1*, thereby increasing the accumulation of the CAT1 protein. Moreover, oxidative stress further promotes the binding of Swi6B to the *CAT1* promoter and the accumulation of the CAT1 protein to counteract excessive ROS accumulation.

### CAT1 improves H_2_O_2_ stress tolerance

**Fig 4:**
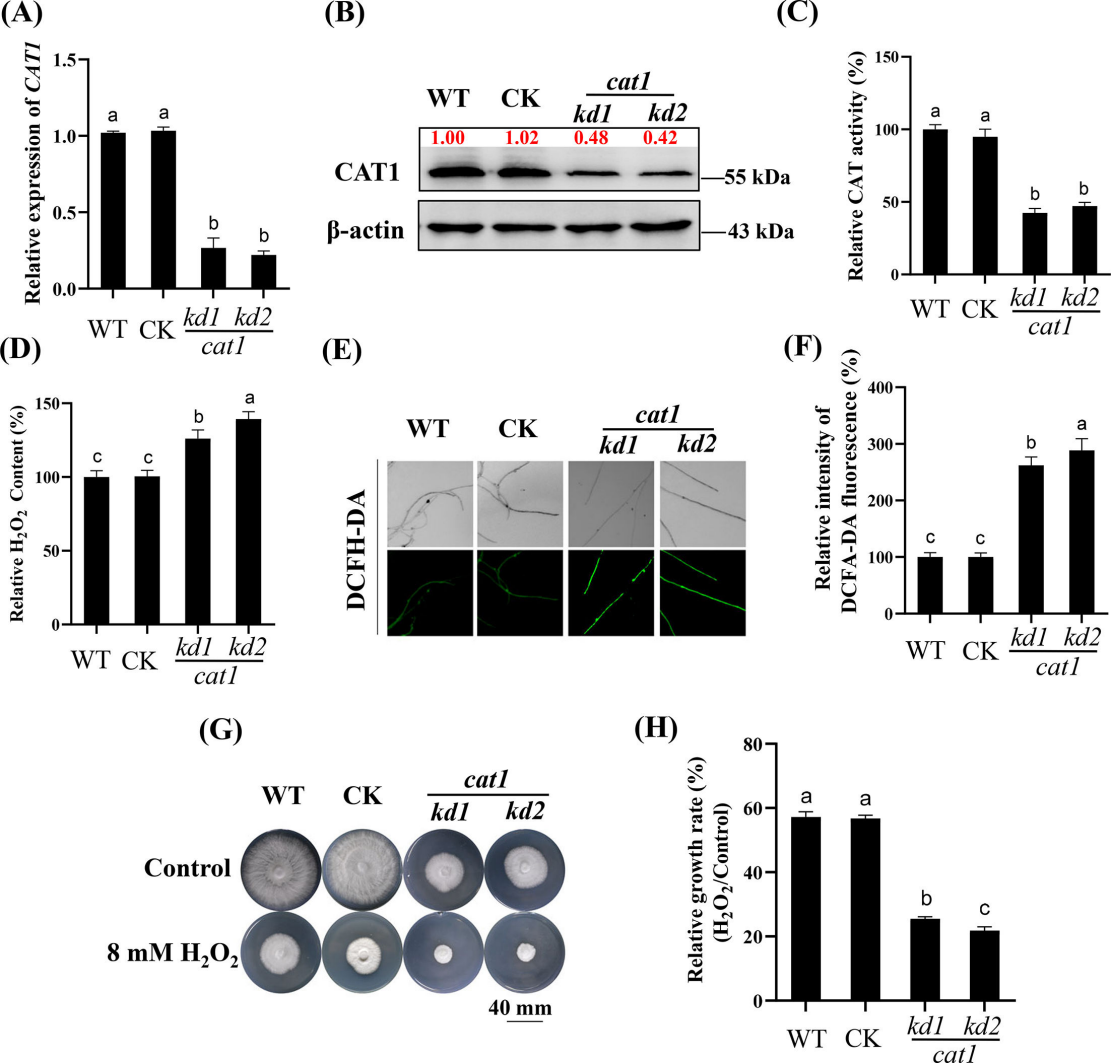
Knockdown of *CAT1* results in enhanced H_2_O_2_ accumulation and reduced tolerance to H_2_O_2_ treatment. (**A and B**) Analysis of the transcription level of the *CAT1* gene by qRT-PCR (**A**) or the abundance of CAT1 protein by western blotting (**B**) in the strains as indicated. (**C**) Relative CAT activity in WT, CK, and *cat1-kds* strains. The activity values were normalized, and the control was set to 100%. (**D**) Relative H_2_O_2_ content determination of WT, CK, and *cat1-kds* strains. The H_2_O_2_ content values were normalized, and the control was set to 100%. (**E and F**) Representative 2′,7′-dichlorodihydrofluorescein diacetate (DCFH-DA) staining pictures (**E**) and the relative fluorescence intensity (**F**) of ROS in the WT, CK, and *cat1-kds* strains. The fluorescence was monitored using a confocal laser scanning microscope with consistent exposure time, and fluorescence signal intensity was determined using ZEN 2011 SP2 software. Values were normalized, and the WT was set to 100%. (**G and H**) The representative pictures (**G**) and relative growth rates (**H**) of different genotype strains under (8 mM) H_2_O_2_ treatment. All strains grown on CYM solid medium supplemented with or without H_2_O_2_. The relative growth rate of each strain was calculated as the diameter of hyphae growth under H_2_O_2_ treatment divided by that under control condition. For panels **A**, **C, D**,** F**, and **H**, the different letters indicate significant differences according to Duncan’s multiple range test (*P* < 0.05).

While numerous studies have documented the capacity of CAT to eliminate ROS, the physiological function of CAT1 in *G. lucidum* remains ambiguous. To investigate the involvement of *CAT1* in response to oxidative stress in *G. lucidum*, knockdown strains (*cat1-kd1/2*) were generated, and the knockdown was confirmed by the significant reduction in *CAT1* transcription and protein expression (Fig. 4A and B). Compared with that of the WT strain, the activity of CAT was significantly lower in the *cat1-kd1/2* strains (Fig. 4C), whereas the level of H_2_O_2_ was markedly greater (Fig. 4D through F). As a result, the growth of the knockdown strain was notably suppressed (Fig. 4G). To investigate the regulatory role of CAT1 in growth under oxidative stress conditions, the WT strain and the *CAT1* knockdown strains were exposed to H_2_O_2_, which revealed growth suppression after H_2_O_2_ treatment (Fig. 4G). The relative growth inhibition rate of the *CAT1* knockdown strain ranged from 79% to 75%, whereas the relative growth inhibition rate of the WT strains was approximately 44% (Fig. 4H). These findings indicated that the ability of *CAT1* to scavenge H_2_O_2_ is crucial for the growth of *G. lucidum*, especially in an oxidative stress environment.

### Slt2 phosphorylates Swi6B to upregulate *CAT1* and increase H_2_O_2_ tolerance

**Fig 5:**
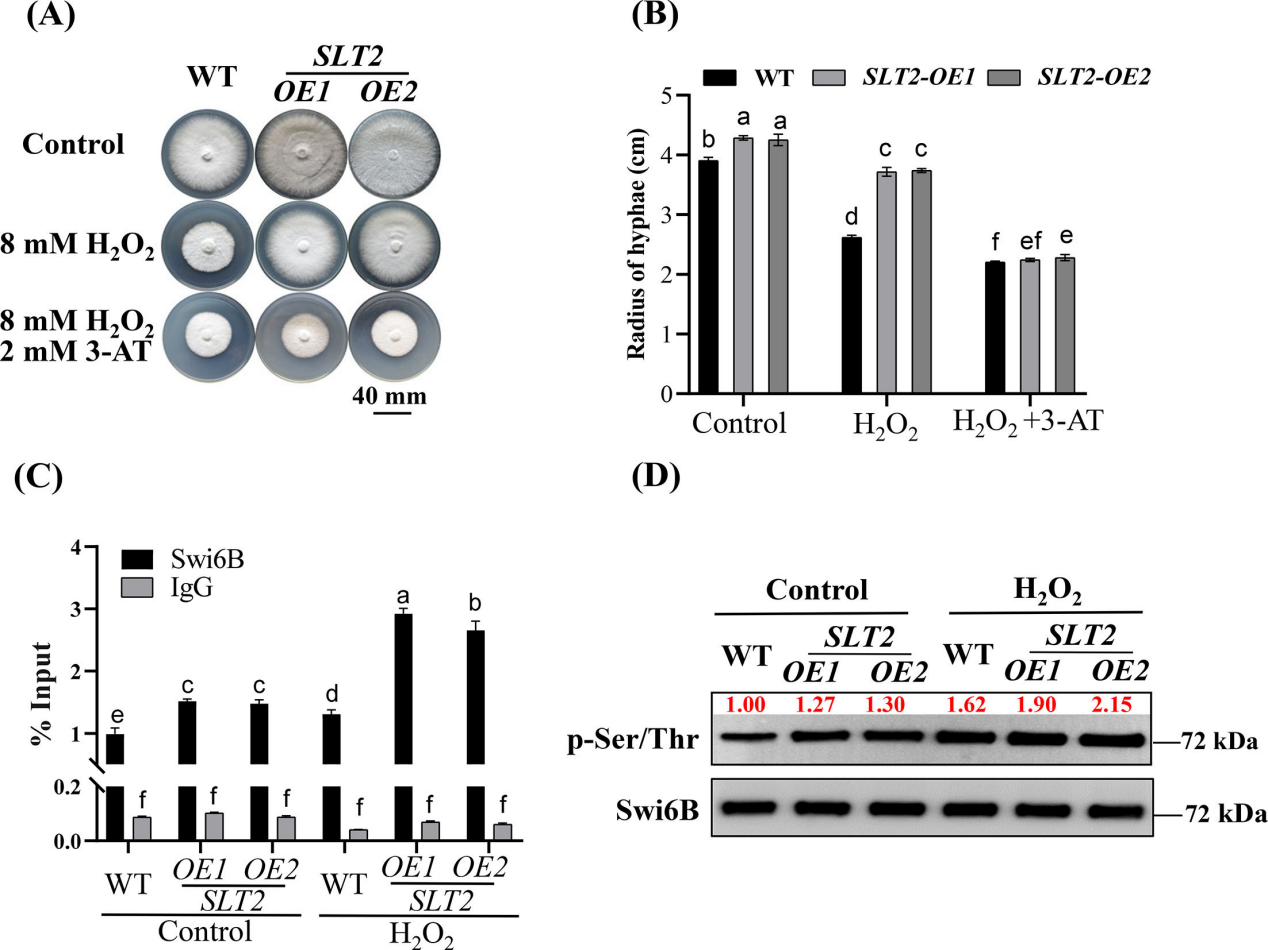
Overexpression of *SLT2* enhances the binding of Swi6B to the *CAT1* by improving the phosphorylation of Swi6B. (**A and B**) The representative pictures (**A**) and hyphae radius (**B**) of *SLT2-OE* and WT strains cultured under different conditions as indicated. (**C**) ChIP-qPCR detection of the enrichment degree of Swi6B on the *CAT1* promoter in the *SLT2-OE* and WT strains grown on CYM solid medium added with or without H_2_O_2_. Immunoprecipitation was performed with anti-Swi6B antibody, and the enriched DNA fragments were used as a template for qPCR. Values were shown as the mean ± SD (*n* = 3) of the cycle threshold. (**D**) Western blotting analysis of the phosphorylation levels of Swi6B in the *SLT2-OEs* and WT strains grown on CYM solid medium added with or without H_2_O_2_. All strains grown on CYM solid medium were added without H_2_O_2_ (8 mM) were set as control. The intensity of bands was analyzed with Image J software (v1.8.0), and the values of WT treated without H_2_O_2_ were set to 1.00. For panels **B** and **C**, the different letters indicate significant differences according to Duncan’s multiple range test (*P* < 0.05).

Previously, we reported that Slt2/Mpk1 (MAPK) interacts with Swi6B in *G. lucidum*, and knockdown of *SLT2* increased the sensitivity of the strain to H_2_O_2_ (17, 28). To further study whether SLT2 is involved in responding to oxidative stress, the expression levels of *SLT2* were detected after H_2_O_2_ treatment. Compared with that of the control, *SLT2* transcription significantly increased after H_2_O_2_ treatment (Fig. S3A). These findings indicated that *SLT2* may be involved in response to oxidative stress. Owing to the identification of the Swi6B-CAT1 axis, we aimed to determine whether Slt2 is also involved in the regulation of *CAT1* through the regulation of Swi6B function in *G. lucidum*. Therefore, we generated *SLT2*-overexpressing strains (*SLT2-OE1/2*; Fig. S3B). Under H_2_O_2_ treatment conditions, the hyphal radius of the *SLT2-OE* strains was greater than that of the WT strain, while the addition of 3-AT resulted in similar growth rates among all the genotypes (Fig. 5A and B). These findings implied that Slt2 may improve resistance to H_2_O_2_ through the regulation of CAT. Therefore, we used ChIP‒qPCR to assess the interaction between Swi6B and the *CAT1* promoter in the WT and *SLT2-OE* strains. Under normal conditions, *SLT2* overexpression increased the binding of Swi6B to *CAT1* (Fig. 5C). Although H_2_O_2_ treatment further enhanced the interaction in all the genotypes, the relative increase rate in the *SLT2-OE* strains (1.80-fold) was obviously greater than that in the WT (1.32-fold; Fig. 5C). Interestingly, the phosphorylation level of Swi6B in the *SLT2-OE* strains (1.27–1.30) was greater than that in the untreated WT strain. Moreover, H_2_O_2_ treatment significantly elevated the Swi6B phosphorylation (1.90–2.10; Fig. 5D), compared with that in the untreated WT strain. However, the relative increase in phosphorylated Swi6B in the *SLT2-OE* strains under H_2_O_2_ treatment was similar to that in the WT strain. The partial dependency of promoter binding on Slt2 activity suggested the involvement of auxiliary regulators, thus warranting further investigation. Therefore, the mechanism by which Slt2 regulates Swi6B phosphorylation under oxidative stress remains to be further investigated in *G. lucidum*. Overall, Slt2 plays a role in response to oxidative stress. In addition, Slt2, which is induced under oxidative stress, further enhances the phosphorylation level of Swi6B, which leads to an increased ability of Swi6B to bind to the *CAT1* promoter, thereby enhancing the oxidative stress tolerance of *G. lucidum*.

## DISCUSSION

Fungi may encounter various stresses during their growth, such as high temperature, drought, and heavy metals, leading to the overproduction of ROS (29, 30). Excessive ROS are harmful to growth and development, and they can even lead to cell death in severe cases (31). Therefore, revealing the mechanism by which fungi respond to ROS bursts to maintain intracellular redox homeostasis is particularly important. CAT is an important component in removing H_2_O_2_ and thus functions as a classic ROS detoxifying enzyme in cells (20). The overexpression of *DhCTA1* improves the tolerance of *Saccharomyces cerevisiae* to oxidative stress (32). In the present study, the knockdown of *CAT1* in *G. lucidum* resulted in reduced growth of the *cat1-kd* strains under optimal conditions (Fig. 4G), which is consistent with the phenotype of *CAT1* knockout strains in *Aspergillus flavus* (20). The decrease in CAT1 level resulted in reduced enzyme activity (Fig. 4A through C). Thus, the *cat1-kd* strains were hypersensitive to H_2_O_2_ (Fig. 4G and H), which is similar to the suppressed tolerance of *Candida albicans* to H_2_O_2_ (33). These results indicated that CAT1 functions as a ROS eliminator in *G. lucidum*, enhancing fungal survival in adverse environments. In addition, the present findings do not rule out additional contributions from other antioxidant enzymes to maintaining redox homeostasis.

Multiple catalases exist in organisms, but they may function under different conditions. Although there are three genes encoding catalase in *Bipolaris oryzae*, only the *CAT2* gene is activated under UV light in the range of 290–400 nm (19). There are also two catalases in *G. lucidum*, while only the transcription and protein levels of CAT1 in the *SWI6B-OE* strains were obviously greater than those in the WT strain, and the *CAT1* transcription and protein levels were lowest in the *swi6-kd* strains (Fig. 2A and D). Notably, H_2_O_2_ treatment significantly increased the enrichment of Swi6B binding to the *CAT1* promoter (Fig. 3C and D) and thus increased *CAT1* transcription and protein levels (Fig. 2A and D), especially in the *SWI6B-OE* strains (Fig. 3D and E). Similarly, CAT1 in *Myxococcus xanthus* is induced rapidly by H_2_O_2_, and the activation of CAT1 is more significant than that in *Streptomyces coelicolor* (34). Consistent with the induced expression of *CAT1*, H_2_O_2_ -induced CAT activity was also highest in the *Swi6B-OE* strains but lowest in the *swi6-kd* strains (Fig. 2B), resulting in the lowest H_2_O_2_ content in the *SWI6B-OE* strains (Fig. 2C). The 3-AT CAT inhibitor completely eliminated the growth advantage of *SWI6B-OE* under H_2_O_2_ treatment (Fig. 4E and F). Together with the *CAT1* promoter binding function of Swi6B (Fig. 3), these results demonstrated that H_2_O_2_-induced CAT1 accumulation partially depends on Swi6B, revealing a novel regulation of *CAT1* gene expression in *G. lucidum*. In *Pseudomonas aeruginosa*, the CzcR transcription factor mediates H_2_O_2_ tolerance in the presence of excess Zn^2+^ by directly binding to the promoter regions of the *katA* and *katB* catalase-encoding genes to inhibit their expression (35). In *Aspergillus flavus*, the SntB epigenetic reader is significantly enriched in the promoter region of *CATC*. After treatment with the menaquinone sodium bisulfite oxidant, the growth and expression of *CATC* have been reported to be more significantly inhibited in the *SntB* deletion strain (36). The hyphal growth of the *ΔMoswi6* mutant is significantly inhibited when it is cultured on media supplemented with H_2_O_2_ (37). In *G. lucidum*, the significant increase in the level of Swi6B caused by the VK_3_ and H_2_O_2_ oxidants also suggested that Swi6B plays an important role in oxidative stress tolerance (Fig. 1C and D). In addition to the reported growth reduction under normal conditions (25), *SWI6* knockdown obviously impaired the tolerance of the WT strain to oxidative stress (Fig. 1A and B). Furthermore, the growth of *G. lucidum* in an oxidative environment was significantly improved by *SWI6B* overexpression (Fig. 1A and B). These data clearly demonstrated that Swi6B is essential for normal growth and plays an important role in enhancing the oxidative stress tolerance of *G. lucidum*. Similarly, FvSwi6 plays an important role in various stress responses during the vegetative growth of *Fusarium verticillioides* by regulating the expression of genes related to carbon metabolism, oxidative stress, and MAPK (38). In *Arthrobotrys flagrans*, AfSwi6 regulates mycelial growth, pathogenicity, and the stress response by regulating the expression of genes related to DNA repair, stress response, and other genes (39). The role of Swi6B in regulating *CAT1* expression could be the mechanism by which Swi6B improves the stress tolerance of *G. lucidum*. However, owing to technical limitations in the generation of secondary genetically modified strains, we cannot generate *SWI6B*-overexpressing strains in the *cat1-kd* background or double mutants with knockdown of both *SWI6* and *CAT1*, preventing elucidation of the genetic relationship between Swi6B and *CAT1*.

One of the important signaling pathways involved in response to oxidative stress is the MAPK family. High concentrations of ROS cause phosphorylation of MAPK and activate the MAPK pathway, which in turn mediates the regulation of ROS by phosphorylating target proteins (40). In *Bipolaris oryzae*, knockout of the *SRM1* MAPK reduces the levels of CAT2, leading to hypersensitivity of the strain to H_2_O_2_ (19). In the present study, the insensitivity of *SLT2-OE* strains to H_2_O_2_ was completely eliminated by the 3-AT CAT inhibitor (Fig. 5A and B), suggesting that Slt2 improves tolerance to oxidative stress predominantly via CAT. Interestingly, Swi6 is a classic target of MAPK in various species (41, 42), and the interaction between Slt2 and Swi6B has also been reported in *G. lucidum* (28). Despite no obvious change in Swi6B level, the phosphorylation level of Swi6B in the *SLT2-OE* strains was greater than that in the WT strain (Fig. 5D). Moreover, regardless of the strain, the level of Swi6B bound to the *CAT1* promoter was also positively correlated with its phosphorylation level of Swi6B (Fig. 5C). These findings suggest that Slt2 promotes the transcription of *CAT1* by increasing the phosphorylation of Swi6B, thereby helping increase the oxidative stress resistance of fungi to oxidative stress.

In summary, we evaluated the function of the APSES transcription factor Swi6B in oxidative stress adaptation in fungi. This study identified CAT1 as a downstream target gene of Swi6B and preliminarily revealed that Slt2 enhances the transcriptional activity of Swi6B under oxidative stress conditions. These findings advance the understanding of the crosstalk between MAPK signaling and fungal antioxidant gene regulation, although comprehensive elucidation of the complete molecular mechanism requires further investigation. The Slt2-Swi6B-*CAT1* axis may be involved in the regulation of oxidative stress tolerance in *G. lucidum*, but the universality of this axis in fungal stress survival needs further validation. In addition to the mechanistic insights of this pathway, it holds significant biotechnological promise. *G. lucidum* contains more than 300 different pharmacologically active compounds, and oxidative stress frequently limits its yield and scalability. The Slt2-Swi6B-*CAT1* axis may play a promoting role in enabling *G. lucidum* to be a cell factory for the production of such compounds. However, more experimental evidence is needed to determine whether Slt2 directly or indirectly phosphorylates Swi6B under oxidative stress. This is an interesting research topic warranting further exploration.

## MATERIALS AND METHODS

### Strains and culture conditions

The *G. lucidum* (ACCC53264) strain from the Agricultural Culture Collection of China was cultured as the WT strain. The construction of *SWI6* knockdown strains (*swi6-kd1/*2) and overexpression strains (*SWI6A-OE1/2 and SWI6B-OE1/2*) was performed previously (26, 27). CYM medium (2% glucose, 1% maltose, 0.2% yeast extract, 0.2% tryptone, 0.05% MgSO_4_ 7H_2_O, and 0.46% KH_2_PO_4_) was used to cultivate the strains. Exogenously added 8 mM H_2_O_2_ (Sinopharm Chemical Reagent Co., Ltd, b3-2-n) or 8 mM VK_3_ (Sangon Biotech, A502486) was used to induce oxidative stress. A 2 mM 3-AT (Sangon Biotech, A601149) was used as the catalase inhibitor. To determine the growth rates of the strains under oxidative stress, all strains were cultured on CYM solid media supplemented with or without chemicals as indicated for 7 days at 28°C. The growth ratios are represented as the diameters of the hyphae. The relative growth ratio was defined as the diameter of the strain under oxidative stress divided by the diameter of the control strain.

### Total RNA extraction and quantitative real-time PCR

Total RNA was extracted with RNA-easy Isolation Reagent (Vazyme, R701-01) from different strains cultured on CYM solid medium supplemented with or without H_2_O_2_ for 7 days at 28°C. The total RNA was subjected to genome DNA removal and first-strand cDNA synthesis via HiScript III All-in-one RT SuperMix Perfect for qPCR (Vazyme, R333-01). qRT-PCR was performed on the fluorescent quantitative PCR instrument (Eppendorf) with AceQ qPCR SYBR Green Master Mix (Vazyme, Q121‒02). The 2^−ΔΔCT^ method was used to determine the relative transcription levels of genes. 18S rRNA was used as an internal reference gene. All primers used are listed in Table S1. The raw data from the qPCR experiments are listed in Table S2.

### Generation of knockdown strains

*CAT1* knockdown strains were constructed via RNA interference (RNAi) technology. The pMi vector was used to construct knockdown strains of *CAT1* (43). The specific DNA fragment of *CAT1* was amplified with the *CAT1*-kd-F/R primers (Table S1) and cloned and inserted into the pMi vector (pMi-*CAT1*). Next, the pMi-*CAT1* vector was introduced into *G. lucidum* via liposome-mediated transfection, and hygromycin resistance was used for the preliminary screening of *CAT1* knockdown strains (44). qRT-PCR and western blot analysis were used to further detect the efficiency of the knockdown strain, and the two strains with the highest knockdown efficiency were used for subsequent experiments.

### H_2_O_2_ content and enzyme activity assay

H_2_O_2_ reacts with titanium sulfate to form a titanium peroxide complex, which exhibits a maximum absorption peak at 415 nm. An H_2_O_2_ content kit (Solarbio, BC3590) was used to determine the H_2_O_2_ content. All the tested strains were cultured at 28°C for 7 days on CYM solid medium with or without H_2_O_2_. Then, 0.1 g mycelium was ground into a powder and subsequently homogenized with 1 mL of H_2_O_2_ extraction buffer. After reacting the homogenate with a working solution containing titanium sulfate, the absorbance at 415 nm was measured.

Samples for CAT activity determination were obtained as described above. Determinations were performed according to the manufacturer's instructions (Solarbio, BC0200). H_2_O_2_ has an absorption peak at 240 nm, and CAT activity represents the rate of decrease in absorbance at 240 nm of H_2_O_2_ per unit time. The strains to be evaluated were cultured according to the above conditions. Briefly, 0.1 g mycelium was ground into a powder and subsequently homogenized with 1 mL of CAT extraction buffer. The homogenate was centrifuged at 8,000 × *g* for 10 min at 4°C, and the supernatant was collected for subsequent analysis. One milliliter of a working solution containing H_2_O_2_ was placed in a 1 mL quartz cuvette, and 35 µL of crude enzyme solution was added, followed by immediate measurement of absorbance at 240 nm (Δ1) and the absorbance after 1 min of reaction (Δ2). The following formulas were utilized to determine CAT activity: ΔA = Δ1 − Δ2 and CAT (U/mg prot) = 678 × ΔA/0.1 g.

The samples used for SOD activity determination were obtained as described above. Determinations were carried out according to the manufacturer’s instructions (Solarbio, BC0170). SOD is a superoxide anion (O_2_^−^) scavenging enzyme. O_2_^−^ reduces nitro blue tetrazolium to form blue formazan, which has a maximum absorption peak at 560 nm. Briefly, the extraction method for SOD crude enzyme solution was the same as that for CAT. One milliliter of a working solution containing O_2_^−^ was placed in a 1 mL quartz cuvette, and 90 µL of crude enzyme solution was added, followed by immediate measurement of the absorbance at 560 nm.

Samples for APX activity determination were obtained as described above. Determinations were performed according to the manufacturer's instructions (Solarbio, BC0220). APX utilizes ascorbic acid (AsA) as an electron donor to catalyze the decomposition of H_2_O_2_ into H_2_O while simultaneously oxidizing AsA to monodehydroascorbate. The activity of APX was measured by detecting the rate of decrease in the absorbance of AsA over 1 min at 290 nm. Briefly, 0.1 g mycelium was ground into a powder and subsequently homogenized with 1 mL of APX extraction buffer. The homogenate was centrifuged at 13,000 × *g* for 20 min at 4°C, and the supernatant was collected for subsequent analysis. A total volume of 900 µL of a working solution was added to a 1 mL quartz cuvette in sequence, and 100 µL of crude enzyme solution was added, followed by immediate measurement of the absorbance at 290 nm for 10 s and 130 s. One unit of APX activity was defined as the oxidation of 1 µmol AsA per gram of tissue per minute.

### ROS detection assay

The intracellular ROS concentration was measured according to a previously described method (45). Intracellular reactive oxygen species oxidize the non-fluorescent 2′,7′-dichlorodihydrofluorescein into the fluorescent 2′,7′-dichlorofluorescein (DCF). By detecting the fluorescence of DCF, the level of reactive oxygen species in *G. lucidum* can be determined. All the tested strains were inoculated on CYM solid medium supplemented with sterile glass coverslips and cultured at 28°C for 7 days. All the samples were incubated with 2′,7′-dichlorodihydrofluorescein diacetate (Beyotime Biotechnology, S0033M, 1:1,000) at 28°C for 20 min. The samples were then washed with deionized water and measured with a Zeiss Axio Imager A1 fluorescence microscope. The excitation wavelength was 488 nm, and the emission wavelength was 525 nm. The intensity of DCF fluorescence represents the content of intracellular ROS. ZEN lite (Zeiss software) was used to analyze the mean fluorescence intensity.

### Y1H assay

The full-length coding gene for *SWI6B* was amplified and inserted into the pGADT7 vector (pGADT7-Swi6B). The specific binding of Swi6B to the DNA fragment of the *CAT1* promoter, including the MCB-binding element (ACGCGT) and the corresponding mutant fragment, was cloned into pAbAi vector (pAbAi-CAT1 and pAbAi-CAT1-M, respectively). First, the pAbAi-CAT1 or pAbAi-CAT1-M vector was transformed into Y1HGold strains grown in uracil-absent SD solid medium (SD/-Ura) for 3–5 days at 30°C. The pGADT7-Swi6B or pGADT7 vector was subsequently transformed into the Y1HGold strains containing the pAbAi-CAT1 or pAbAi-CAT1-M vector, which were subsequently grown on solid leucine-absent SDs medium supplemented with 300 ng/mL aureobasidin A (AbA; SD/-Leu/+AbA).

### Electrophoretic mobility shift assay

The full-length coding gene for *SWI6B* was cloned and inserted into the pET-32a vector (pET-32a-Swi6B) with a His-Trx tag and expressed in BL21 *Escherichia coli* strain (DE3) to obtain the His-Trx-Swi6B protein with Ni-charged MagBeads (GenScript). DNA fragments from the promoter region of *CAT1* and mutant fragments were amplified using primers that were either biotin-labeled at the 5' end or unlabeled with a probe. Swi6B protein was incubated with different probes at 30°C for 30 min. Western blot analysis was used to detect the specific binding of Swi6B to the DNA fragment of the *CAT1* promoter. All the procedures used were performed according to the manufacturer’s specifications (Thermo Scientific, 89880). All primers used are listed in Table S1.

### ChIP-qPCR analysis

By recognizing and binding to the intracellular Swi6B protein in *G. lucidum* through the anti-Swi6B antibody, the DNA fragments associated with it were enriched, and subsequently, the sequences of these DNA fragments and their binding with Swi6B were analyzed by qPCR. All the samples used for ChIP-qPCR were obtained as described above. The ChIP assay was performed according to a previously established method (46). Briefly, 2.0 g mycelia were incubated with 1% formaldehyde for 25 min to cross-link proteins to DNA. Nuclei were isolated from liquid nitrogen-ground samples and prepared by sonication to obtain sheared chromatin (250−500 bp). Polyclonal antibodies against Swi6B and negative control rabbit serum (Sangon Biotech, D601019) were then used to immunoprecipitate the sheared chromatin. At the same time, samples removed in advance and not immunized with antibodies were used as inputs. qPCR was performed to detect the binding efficiency of Swi6B to the *CAT1* promoter. The enrichment values were normalized to those of the input sample. All primer sequences used for ChIP-qPCR are shown in Table S1.

### *In vivo* phosphorylation assay

To detect the phosphorylation level of Swi6B in *G. lucidum*, co-immunoprecipitation was used to obtain the endogenous Swi6B protein. In brief, all the strains were cultured on CYM solid medium with or without 8 mM H_2_O_2_ for 7 days at 28°C. Immunoprecipitation lysis buffer (100 mM NaCl, 20 mM Tris-HCl pH 7.5, 1 mM dithiothreitol (DTT), 10% glycerol, 1 mM phenylmethylsulfonyl fluoride (PMSF), and protease inhibitor cocktail) was used for extraction of total protein of *G. lucidum*. The total protein was first incubated with anti-Swi6B antibody at 4°C for 8 h. Then, protein A/G magnetic beads (Thermo Fisher Scientific, 88802) were added to the sample mixture and incubated at room temperature for 1 h. Finally, the Swi6B proteins were eluted from the beads with SDS sample buffer (50 mM Tris-HCl pH 6.8, 1% SDS; 10% glycerol; 50 mM DTT; and 0.005% bromophenol blue). Western blotting assay was used to evaluate the phosphorylation levels of Swi6B in different strains with anti-pSer/Thr (1:4,000, Abcam, ab117253) and anti-Swi6B (1:2,000, rabbit polyclonal antibody) antibodies.

### Western blotting assay

For total protein extraction, the strains to be assayed were cultured on CYM solid medium supplemented with or without H_2_O_2_ for 7 days at 28°C. The mycelia of *G. lucidum* from different treatments were ground in liquid nitrogen to obtain total protein. Western blot analysis was performed according to a previous method (44). Specific anti-Swi6B (1:2,000, rabbit polyclonal antibody), anti-CAT1 (1:2,000, rabbit polyclonal antibody), anti-pSer/Thr (1:4,000, Abcam, ab117253), and anti-β-actin (1:2,000, Abcam, M20011) were used for western blotting assay. The band intensities were analyzed with ImageJ software (v1.8.0). The ratio of target protein to actin grayscale in the WT was set to 1.00 for normalization.

### Statistical analysis

Statistical analysis was performed according to Duncan’s posttest, and GraphPad Prism 8 (GraphPad Software, Inc.) was used to perform the statistical analysis in this study (43). The results measurements from at least three independent samples were averaged, and the results are shown as the means ± SDs. Data analysis by one-way analysis of variance with Duncan’s posttest was used for multiple comparisons, and different letters correspond to *P* < 0.05.

## Data Availability

The authors confirm that the data supporting the findings of this study are available within the article and its supplemental material.

## SUPPLEMENTAL MATERIAL

Fig. S1 aem.00679-25-s0001.tif Swi6A does not respond to H_2_O_2_ and has no function on tolerance to H_2_O_2_ treatment.

Fig. S2 aem.00679-25-s0002.tif Fold change of enzyme activity assay of APX and SOD in different strains cultured under control and H_2_O_2_ treatment conditions.

Fig. S3 aem.00679-25-s0003.tif Detection of SLT2 expression levels.

Supplemental legends aem.00679-25-s0004.docx Legends for Fig. S1 to S3.

Table S1 aem.00679-25-s0005.docx Primers used for gene expression analysis.

Table S2 aem.00679-25-s0006.xlsx Raw data from the qPCR experiments.

ASM does not own the copyrights to Supplemental Material that may be linked to, or accessed through, an article. The authors have granted ASM a non-exclusive, world-wide license to publish the Supplemental Material files. Please contact the corresponding author directly for reuse.
