# Supplementary figures and images for: The APSES transcription factor Swi6B upregulates CATALASE 1 transcription to enhance oxidative stress tolerance of Ganoderma lucidum
Source: Appl Environ Microbiol. 2025 Jun 18;91(7):e00679-25. doi: 10.1128/aem.00679-25 (PMC12285235; doi:10.1128/aem.00679-25)

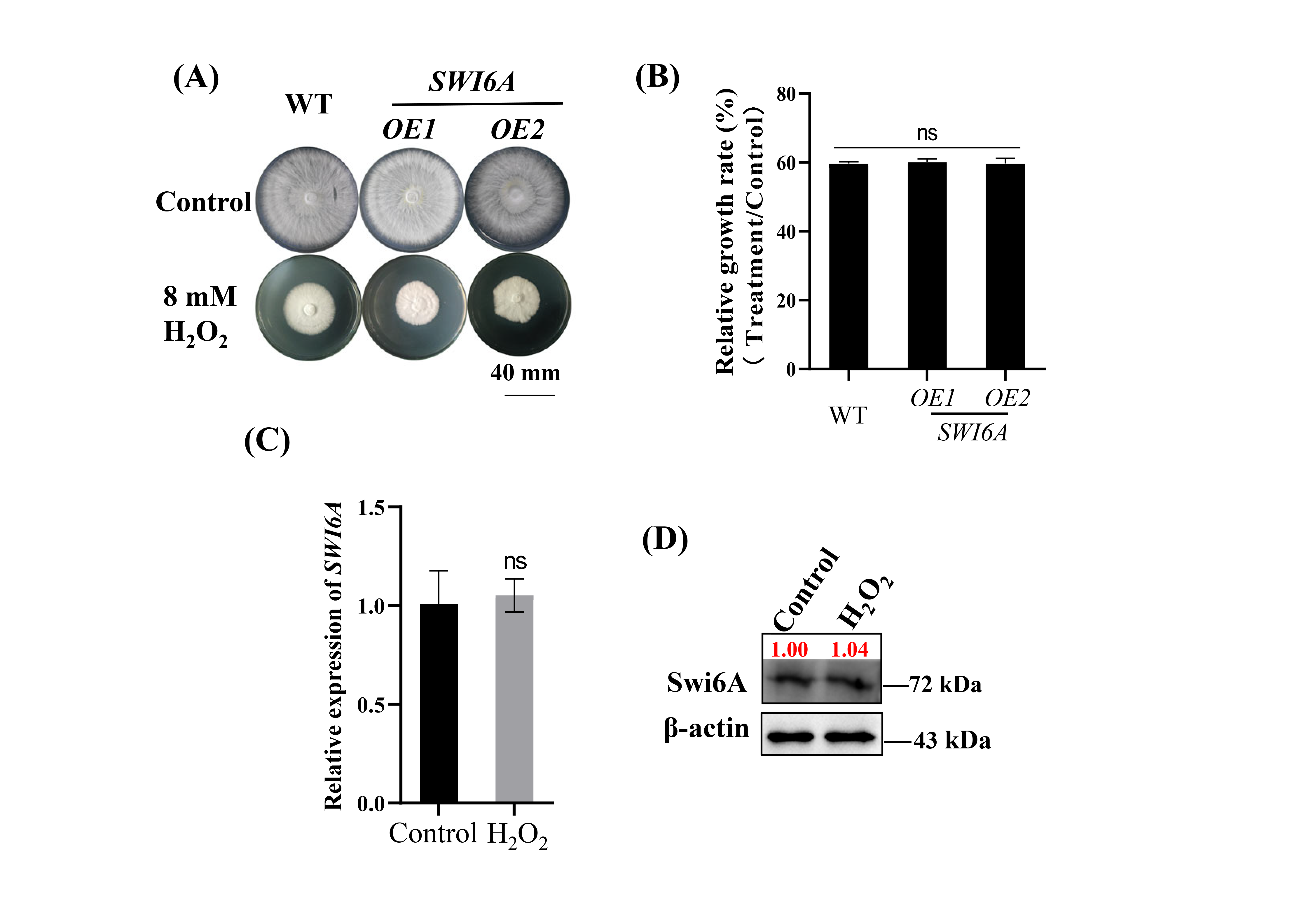

Supplement: Fig. S1 — Swi6A does not respond to H2O2 and has no function on tolerance to H2O2 treatment. [file aem.00679-25-s0001.tif]

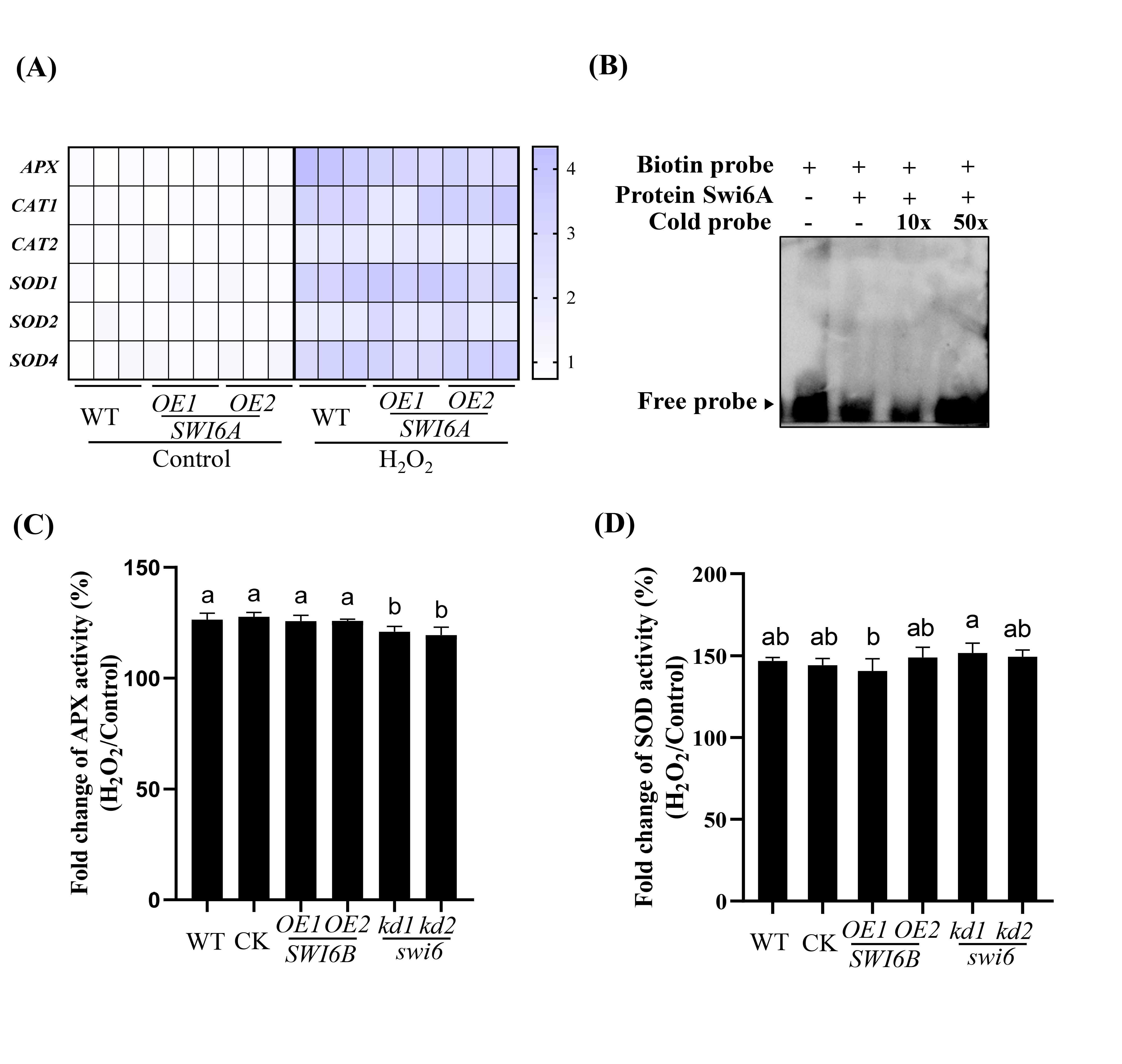

Supplement: Fig. S2 — Fold change of enzyme activity assay of APX and SOD in different strains cultured under control and H2O2 treatment conditions. [file aem.00679-25-s0002.tif]

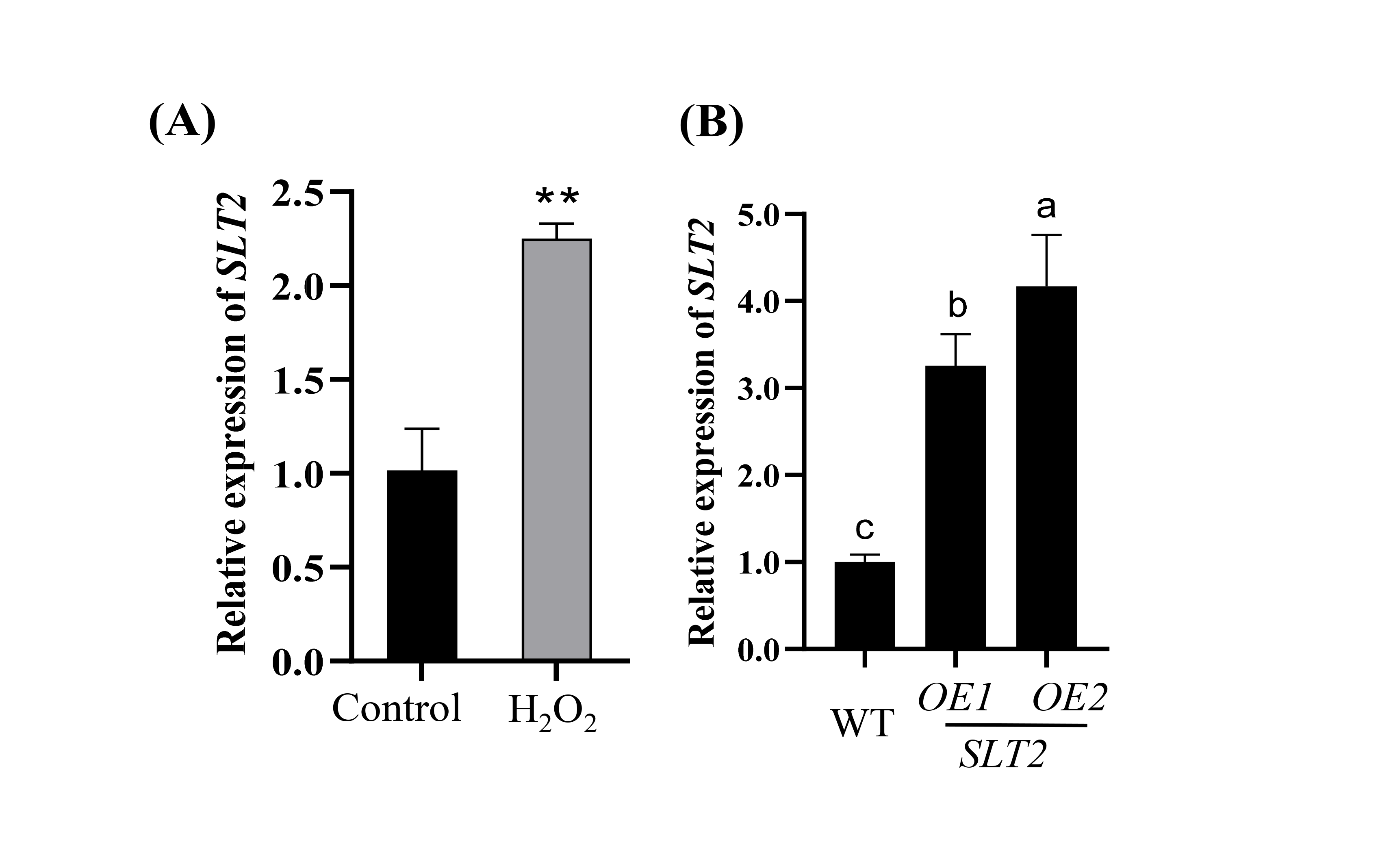

Supplement: Fig. S3 — Detection of SLT2 expression levels. [file aem.00679-25-s0003.tif]
