# Supplementary material for: The APSES transcription factor Swi6B upregulates CATALASE 1 transcription to enhance oxidative stress tolerance of Ganoderma lucidum
Source: Appl Environ Microbiol. 2025 Jun 18;91(7):e00679-25. doi: 10.1128/aem.00679-25 (PMC12285235; doi:10.1128/aem.00679-25)
Supplement: Supplemental legends — Legends for Fig. S1 to S3. [file aem.00679-25-s0004.docx]

significant differences according to Duncan’s multiple range test (*p* < 0.05).

**Figure S1. Swi6A does not response to H_2_O_2_ and has no function on tolerance to H_2_O_2_ treatment.**

**(A-B)** The represent pictures (A) and relative growth rates (B) of the WT and *SWI6A-OE* strains under H_2_O_2_ treatment. All strains were cultured on CYM solid medium supplement with or without (8 mM) H_2_O_2_. The relative growth rate of each strain was calculated as the diameter of hyphae growth under H_2_O_2_ treatment divided by that under control condition. The data was presented as percentage. *SWI6A-OEs*, SWI6A overexpression strains; CK, empty vector strains. **(C)** qRT-PCR analysis of the expression levels of *SWI6B* in the WT strain cultured with or without H_2_O_2_ treatment. **(D)** Western blotting analysis of the Swi6A protein in the WT strain grown on CYM solid medium supplemented with or without H_2_O_2_. The intensity of bands was analyzed with Image J software (v1.8.0). For (B) and (C), the significant differences were tested according to Duncan’s multiple range test and no significant differences was found.

**Figure S2.** **Fold change of enzyme activity assay of APX and SOD in different strains cultured under control and** **H_2_O_2_ treatment condition.**

**(A)** Heat map of the expression levels of genes encoding classical antioxidant enzymes with or without H_2_O_2_ treatment. The expression levels of genes in all the genotypes were analyzed by RT–qPCR. **(B)** EMSA assays exhibited that Swi6A does not interact the *CAT1* promoter region. **(C)** Fold change in the relative APX activity in the *SWI6B-OE*, *swi6-kd*, WT and CK strains. **(D)** Fold change in the relative SOD activity in the *SWI6B-OE*, *swi6-kd*, WT and CK strains. The relative enzyme activity (%) of different strains in the presence of H_2_O_2_ treatment was calculated as the activity containing (8 mM) H_2_O_2_ divided by the control. The different letters indicate significant differences according to Duncan’s multiple range test (*p* < 0.05).

**Figure S3.** **Detection of *SLT2* expression levels.**

**(A)** Analysis of the transcription level of *SLT2* in the *SLT2-OE* and WT strains by qRT-PCR. **(B)** qRT-PCR analysis of the expression levels of *SLT2* in the WT strain cultured with or without H_2_O_2_ treatment. For (A-B), Values ​​are shown as the mean ± SD (n=3), the statistical significance is conducted with Student's t test. (*, P < 0.05; **, P < 0.01).
