# Supplementary material for: The APSES transcription factor Swi6B upregulates CATALASE 1 transcription to enhance oxidative stress tolerance of Ganoderma lucidum
Source: Appl Environ Microbiol. 2025 Jun 18;91(7):e00679-25. doi: 10.1128/aem.00679-25 (PMC12285235; doi:10.1128/aem.00679-25)
Supplement: Table S1 — Primers used for gene expression analysis. [file aem.00679-25-s0005.docx]

**Table S1.** Primers used for gene expression analysis

| Primer | Sequence (5’ to 3’) | Description |
| --- | --- | --- |
| RT-*18S*-F | TATCGAGTTCTGACTGGGTTGT | Detects the *18S* expression |
| RT-*18S*-R | ATCCGTTGCTGAAAGTTGTAT |  |
| RT-*APX*-F | TTTTCGGTGCCCTTGGTGC | Detects the *APX* expression |
| RT-*APX*-R | GTAGTTCTTATCAGCCGCCT |  |
| RT-*CAT1*-F | TACGGTATTCAGCTCTTGT | Detects the *CAT1*expression |
| RT-*CAT1*-R | TGTTGTAACGGAACTTCTC |  |
| RT-*CAT2*-F | GATTTGGCGTCAACACTTT | Detects the *CAT2* expression |
| RT-*CAT2*-R | ACTCGACATAACCTCCTTCC |  |
| RT-*SOD1*-F | ATCGCCGTCTTCGTCGTTT | Detects the *SOD1* expression |
| RT-*SOD1*-R | GTGACTGGTGCGGTAGGGA |  |
| RT-*SOD2*-F | AATGCTGGAGCGTGCTGGGTC | Detects the *SOD2* expression |
| RT-*SOD2*-R | CGGATGCGTATGGAGTGGTC |  |
| RT-*SOD4*-F | CTCACCGCGACGCGATTAC | Detects the *SOD4*expression |
| RT-*SOD4*-R | CCCTTGCCCTCAGACTTGG |  |
| RT-*SWI6B*-F | CCCCATCTACATCCACAGCTG | Detects the *SWI6B* expression |
| RT- *SWI6B*-R | CGCGATCGGTCCAGCATT |  |
| RT-*SWI6A*-F | TGTTTCACCTGGCCCACA | Detects the *SWI6A* expression |
| RT-*SWIA6*-R | CGCGATCGGTCCAGCATT |  |
| *PCAT1*-pABAi-F | AAGCTTTCGCTTCAACGCCTCACGCGTAC | *CAT1* promoter fragment for YIH validation. |
| *pCAT1*-pABAi-R | TTGATCGTACGCGTACAGCTG |  |
| Swi6B-AD-F | GAATTCATGTGGCTCATGTTCGCACACAGG | Get Swi6B for construction of AD-Swi6B |
| Swi6B-AD-R | CTCGAGCTATATTACACCGTTTCGTACCTTCTGCATG |  |
| *pCAT1*-Biotin-F | TCAACGCCTCACGCGTACGCCAAG | Biotin-labeled *SWI6* promoter fragment for EMSA validation. |
| *pCAT1*-Biotin-R | ACCACGGTCAGCTGTACGCGTAC |  |
| *pCAT1*-M-Biotin-F | TCAACGCCTCAAAAAAACGCCAAG | Biotin-labeled *SWI6* promoter mutant fragment for EMSA validation. |
| *pCAT1*-M-Biotin-R | TTGATCGTAAAAAAACAGCTG |  |
| *pCAT1*-F | TCGCTTCAACGCCTCACGCGTAC | Competing fragment of *SWI6* promoter for EMSA validation. |
| *pCAT1*-R | ACCACGGTCAGCTGTACGCGTAC |  |
